# A Surface Plasmon Resonance-Based Optical Fiber Probe Fabricated with Electropolymerized Molecular Imprinting Film for Melamine Detection

**DOI:** 10.3390/s18030828

**Published:** 2018-03-09

**Authors:** Wei Li, Yongping Zheng, Tingwei Zhang, Songjie Wu, Jue Zhang, Jing Fang

**Affiliations:** 1 Academy for Advanced Interdisciplinary Studies, Peking University, Beijing 100871, China; liwei43@pku.edu.cn (W.L.); tingweiz@pku.edu.cn (T.Z.); jfang@pku.edu.cn (J.F.); 2 College of Engineering, Peking University, Beijing 100871, China; john_zyp@pku.edu.cn (Y.Z.); sjwu@pku.edu.cn (S.W.)

**Keywords:** surface plasmon resonance, electropolymerization, optical fiber probe, molecular imprinting, melamine

## Abstract

Molecularly imprinted polymer (MIP) films prepared by bulk polymerization suffer from numerous deficiencies, including poor mass transfer ability and difficulty in controlling reaction rate and film thickness, which usually result in poor repeatability. However, polymer film synthesized by electropolymerization methods benefit from high reproducibility, simplicity and rapidity of preparation. In the present study, an Au film served as the refractive index-sensitive metal film to couple with the light leaked out from optical fiber core and the electrode for electropolymerizing MIP film simultaneously. The manufactured probe exhibited satisfactory sensitivity and specificity. Furthermore, the surface morphology and functional groups of the synthesized MIP film were characterized by Atomic Force Microscopy (AFM) and Fourier transform infrared microspectroscopy (FTIR) for further insights into the adsorption and desorption processes. Given the low cost, label-free test, simple preparation process and fast response, this method has a potential application to monitor substances in complicated real samples for out-of-lab test in the future.

## 1. Introduction

Melamine (2,4,6-triamino-1,3,5-triazine, MEL), a triazine analog with three amino groups, is widely used in the production of melamine formaldehyde resins for surface coatings, laminates, and adhesives [1]. Containing a substantial amount of nitrogen (66.7%) by mass, MEL has been illegally added to dairy products to boost the apparent total nitrogen content reading. Melamine combined with cyanuric acid can cause renal failure in human beings and animals due to precipitation of insoluble crystals of melamine–cyanurate adduct in the kidneys [2,3,4]. In 2004 and 2007 large outbreaks of nephrotoxic renal failure occurred in dogs and cats that were attributed to overdosing on melamine in adulterated pet food [4,5]. In September 2008, infant formulas illegally adulterated with MEL were found, which caused thousands of infants in China to suffer from kidney stones with subsequent renal failure and death if the infants were not treated [6,7,8]. Standard limits of 1 ppm (8 mM) for MEL in infant formula and 2.5 ppm (20 mM) in other milk products have been introduced by many countries [9,10]. In Canada, the maximum levels of melamine allowed in dried infant formula and other products containing milk are 0.5 ppm and 2.5 ppm, respectively [2]. Therefore, determination of melamine is of biological, clinical, and food industry importance.

Numerous instrument analytical methods have been employed for the detection of melamine, such as gas chromatography (GC) [11], high performance liquid chromatography (HPLC) [12], hyphenated mass spectrometry methods [5,13,14,15,16,17,18,19], surface enhanced Raman spectroscopy (SERS) [20], near-infrared spectroscopy (NIR)/mid-infrared spectroscopy (MIR) [21], nuclear magnetic resonance spectroscopy (NMRS) [22], enzyme-linked immunosorbent assay (ELISA) [23]. Fourier transform infrared (FTIR) spectroscopy [21], electrochemiluminescence [24], and chemiluminescence [25]. These methods provide accurate and sensitive ways to detect melamine, however, these expensive large-size instruments are less suitable for field detection situations. Furthermore pretreatment including extraction, preconcentration or derivatization complicate the detection process [10]. It is desirable to establish a portable, low cost and quick method to detect MEL.

The molecular imprinting technique provides a convenient, low cost approach to synthesize polymer matrices with binding sites for molecular recognition, which are complementary in shape and size related to the templates, showing a promising future in high specificity sensing. There are many forms of molecularly imprinted polymers (MIPs), mainly including bulks, microspheres and films [26]. The most conventional approach is to synthesize MIPs in bulk, then grind and sieve the particles into desired size range according to the specific application. Irregular particles in size and shape were obtained and partial binding sites are destroyed during grinding, reducing the MIPs’ rebinding ability. In recent years, MIP films synthesized by the method of in situ polymerization have attracted significant interest due to the convenience in controlling the thickness and porosity [27,28], which enable many applications, such as electrochemical sensor [29,30], quartz crystal microbalance (QCM) [31], and capillary electrochromatography [32].

In recent decades, surface plasmon resonance (SPR) technologies have flourished in a wide range of applications such as chemical sensors and biosensors, especially in applications that demand highly sensitive, fast responses and label-free detection. The resonance wavelength exhibit an unique response to changes in boundary conditions such as an increase in the effective refractive index near the metal surface [33]. The most common approach to excite SPR is based on the attenuated total reflection method that employs a prism coupler with a thin metal film, which usually referred to as the Kretschmann method [34]. Although prism provide a wonderful coupling interface to create total reflection, optical fiber [35]-based SPR (OF-SPR) sensors possess extraordinary advantages such as low cost, miniaturized sensor systems, simple and flexible design and remote sensing capability [35].

Numerous studies have combined the merits of MIP films and OF-SPR sensors to carry out high specificity detection of analytes [36,37,38,39,40,41,42,43]. However, an overwhelming majority of the extant researches adopt bulk polymerization to synthesize MIP films on the core surfaces of optical fibers. MIP films prepared by bulk polymerization suffer from numerous deficiencies, including: (1) poor mass transfer and rebinding ability, (2) incomplete template removal during MIP preparation, (3) excessively long polymerization times, (4) difficulty in controlling reaction rates and film thicknesses [43,44], which usually result in poor repeatability. Several procedures are used to prepare MIP films through in-situ polymerisation such as spin-coating [45], layer-by-layer deposition [46,47], electropolymerisation [48]. Electropolymerization methods position electroactive functional monomers spatially around the template to create permanent memory elements for rebinding. This procedure enables the possibility to effectively control: (1) the rate of polymer nucleation and growth by the proper selection of electropolymerization parameters, (2) the film thickness by adjusting the amount of charges passed during film electropolymerization, (3) the film morphology by suitable selection of solvent and supporting electrolyte and (4) the adherence to the substrate surface, which result in high reproducibility, simplicity and rapidity of preparation [44]. When the polymer film on the electrode grows thick enough to become resistive, further access of monomer to the electrode surface is limited. Therefore, a typical thickness of electropolymerized film is on the order of dozens of nanometers [49,50]. 

In the present study, an Au film served as the refractive index-sensitive metal film to couple with the light leaked out from optical fiber core and the electrode for electropolymerizing MIP film simultaneously. Based on this proposed concept, we fabricated and characterized OF-SPR probes through the cyclic voltammetry (CV) technique, using *o*-aminophenol as a functional monomer to synthetize MIP films for the detection of melamine. Fourier transform infrared (FTIR) microspectroscopy and atomic force microscopy (AFM) were employed in order to verify the functional groups of MIP films. Different concentrations of melamine solutions were chosen for the characterization of optical fiber probes. The specificity test was carried out against acrylamide, tetracycline, urea, glucose solutions. To the best of our knowledge, we are reporting for the first time the synthesis of electropolymerized MIP film on the surface of OF-SPR probe.

## 2. Materials and Methods

### 2.1. Reagents and Apparatus

Commercial multimode polymer clad silica fiber (NA 0.4, core and cladding diameter 600 μm and 660 μm, respectively) used for the fabrication of the probes, was purchased from Wyoptics (Shanghai, China). Melamine and *o*-aminophenol was used as template molecule and functional monomer, respectively. Phosphate buffered saline (PBS, pH = 7) was prepared using 0.1 mol/L K2HPO4 and 0.1 mol/L KH_2_PO_4_. Methanol, acetonitrile and acetic acid were used for the removal of template molecules from the MIP polymer film. Urea, glucose acrylamide and tetracycline were chosen for the specificity test. All the chemicals used were of analytical grade and were purchased from Sinopharm Chemical Reagent Co., Ltd. (Beijing, China). All aqueous solutions were prepared with purified water (Merck Chemicals (Shanghai) Co., Ltd., Shanghai, China). All electrochemical experiments were carried out at room temperature. High purity nitrogen was purchased from local vendor and was used for deaeration and blow-drying.

Molecular imprinting polymerization was performed with a three-electrode system connected to a CHI 620E electrochemical workstation (Chenhua Instruments Co., Shanghai, China). The actual pH values were determined with a microprocessor pH-meter (LE438, Mettler-Toledo, Shanghai, China). Uniform coating of Cr and Au film were performed inside a magnetron sputtering equipment. Surface topography of the MIP and non-imprinted polymer (NIP) films before and after template removal were carried out with atomic force microscopy (SPA-400, SII NanoTechnology (Shanghai) Inc., Shanghai, China). Functional groups on the surface of MIP films were characterized with FTIR microspectroscopy (Spotlight200, PerkinElmer (Shanghai) Inc., Shanghai, China). Fiber optic probes were characterized by a tungsten halogen lamp (DH2000-BAL, Ocean Optics Asia, Shanghai, China), which exhibits a wavelength emission ranging from 360 nm to 2500 nm and a spectrometer with detection resolution of 0.3 nm (Avaspec-3648, AVANTES B.V., Eerbeek, The Netherlands). To measure the change of normalized transmitted power, the resonance wavelength was obtained through transmittance mode, which follow the equation:

(1) $$T_{n}=100\times \left(sample_{n}-dark_{n}\right)/\left(ref_{n}-dark_{n}\right),$$

where $sample_{n}$ is the current sample value at pixel n, $dark_{n}$ is the value at pixel *n* without exposure to light, $ref_{n}$ is the value at pixel n without any sample.

### 2.2. Probe Fabrication

About 12 cm long fibers were used for the fabrication of different types of probes. Both endfaces were carefully polished. The coated layer of the multimode fibers were unclothed using a sharp blade. Then the cladding of sensing sections (about 13 mm long) were completely removed by immersing into hydrogen fluoride (HF) acid to make the optic power leak out from the core. After a certain time, cleanout the exposed length to keep the etched area smooth. The etching time is related to the optical power leak out and fiber diameter.

Uniform coating of Cr (5 nm) and Au (50 nm) film were performed by fixing the probe into a homemade rotating (along the axis of optical fiber) apparatus inside a magnetron sputtering system. The chromium film served as adhesive layer while the gold film acted as sensitive layer as well as conducting layer. 

### 2.3. Electrochemical Synthesis of MIP

Molecular imprinting polymerization was performed with a three-electrode system. The electrochemical reaction solution was made by the following procedure: o-aminophenol (1.5 mM) was dissolved in 25 mL of prepared PBS solution. Then the pH value was buffered close to 6.8. The imprinted molecule melamine (0.08 mM) and 10mL of MilliQ water were then added into the solution. We transferred this solution to a 50 mL volumetric flask, diluted with acetonitrile to volume, and then agitated with ultrasonic instrument over 5 min to remove oxygen. 

Electrochemical polymerization for MIP probes was performed in a 70 mL volume self-made cylindrical electrolytic cell accommodating three electrodes, using the optical fiber probe coated with Cr/Au film as working electrode. A potassium chloride (KCl)-saturated Ag/AgCl electrode and a platinum plate electrode were used as reference and counter electrodes respectively. The optical fiber probe coated with Cr/Au was then dipped into the reaction solution and incubated for 30 min. The cylindrical electrolytic cell needed to be tightly closed and light blocking.

**Figure 1:**
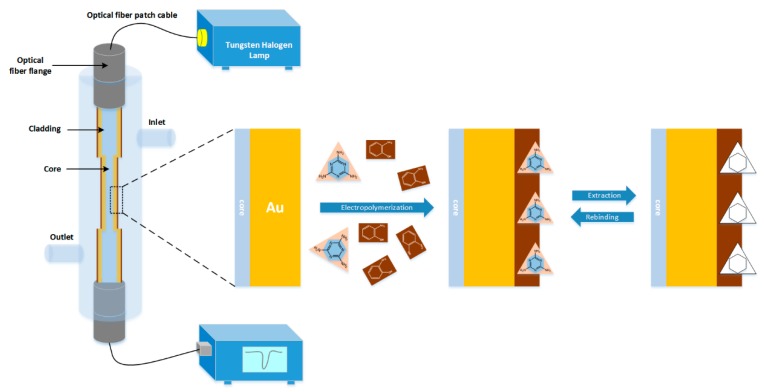
Schematic diagram of the experimental setup and the fabrication process of PoAP–MEL probe.

The electropolymerization was performed by cyclic voltammetry (CV) for 30 cycles in the potential range from −0.3 V to 1.2 V with a scan rate of 50 mV/s. The NIP probes were prepared in the same way but without the addition of template MEL. The removal of the template MEL was carried out by immersing the probes prepared above in desorption solution, an acetic acid: methyl alcohol: acetonitrile (3:4:4) solution for over 4 h at room temperature under continuous agitation. Then the probes were washed with ethanol to remove the template molecules and adsorbates on the surface of the imprinted polymer film, and dried under pure nitrogen flow for further use. The finished imprinted probes were stored at 4 °C in dry condition. A schematic diagram of the probe preparation is shown in Figure 1. 

### 2.4. Experimental Setup

Figure 1 shows a schematic of experimental setup used for the characterization of the sensing probe. The prepared optical fiber probe was fixed in a flow cell with sample inlet and outlet channel on the cylindrical surface so that the sample could interact with the sensing region. Meanwhile, optical fiber splices were set at both endfaces of the flow cell. The flow cell was then connected to the halogen lamp and spectrometer through Optical fiber patch cables and optical fiber flanges for the maximum launching of the light into the probe. SPR response for various concentrations of Melamine samples were recorded immediately after 30 s of incubation. The sensing surface of the fiber was washed with MilliQ water between two consecutive samples.

## 3. Results and Discussion

### 3.1. Electropolymerization of Molecularly Imprinted Film

**Figure 2:**
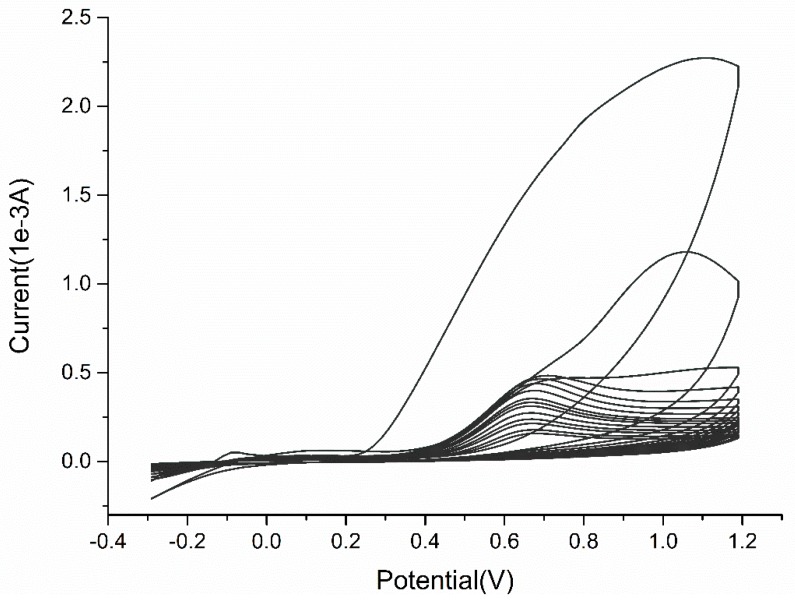
Cyclic voltammogram for the electropolymerization of MIP film on Au surface. Scan rate: 50 mV/s; number of scans: 30; potential range: −0.3 V to 1.2 V.

A typical cyclic voltammogram recorded during electropolymerization in the presence of functional monomer oAP and template MEL under a scan rate of 50 mV/s is shown in Figure 2. It is observed that a distinct irreversible broad anodic oxidation peak appeared at +1.1 V (versus Ag/AgCl) on the anodic branch at the first scan. During successive scans (up to 30 cycles), the peak current decreased and shifted to more negative potentials (stabilized around +0.65 V), which was in accordance with the feature reported by Evrim et al. [51]. Meanwhile a dark brown and homogeneous PoAP film was observed on the gold surface of optical fiber probe. Here, the oxidation peak shifted from +1.1 V to +0.65 V may related to the dimer oxidation to a semi-oxidized state followed by the total-oxidized state process [52]. No reduction peak was observed during the polymerization of oAP. Electropolymerization under a scan rate of 5 mV/s is shown in Figure S1. At a lower scan rate, the oxidation peak stabilized around +0.6 V without peak shifting, which might indicate that a total-oxidized state process directly took place on the gold surface. It did not have significant differences in comparison with the cyclic voltammogram obtained under the same conditions in the absence of MEL template.

### 3.2. FTIR Microspectroscopy of MIP Film

**Figure 3:**
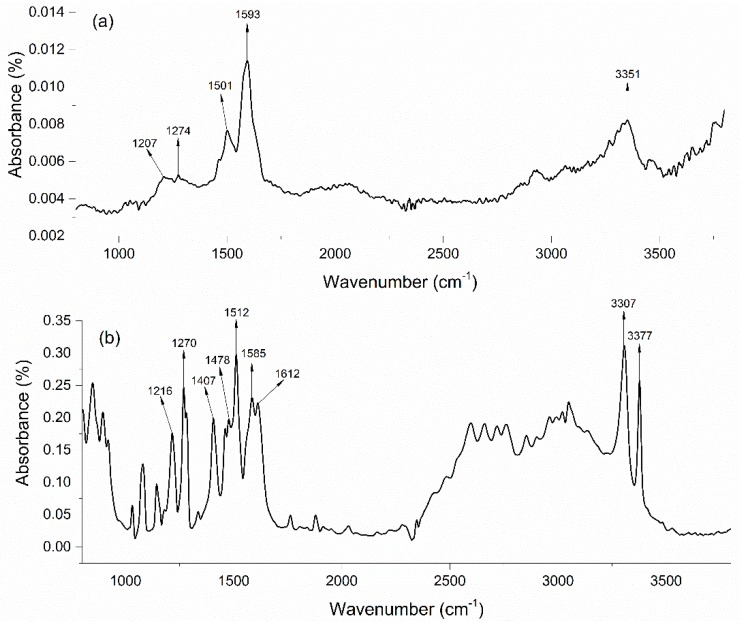
FTIR spectrum of (**a**) PoAP–MEL film (**b**) oAP.

Since the MIP film on the surface of OF-SPR probe was synthesized by cyclic voltammetry (30 cycles) in the potential range from −0.3 V to 1.2 V under a scan rate of 50 mV/s, the MIP film was too thin to generate an infrared signal (data not shown). To characterize the surface groups on PoAP–MEL film, a thicker film sample was synthesized on the surface of a glass slide (1 cm × 1.5 cm) coated with Au film by cyclic voltammetry (150 cycles) in the potential range from −0.4 V to 1.3 V under a scan rate of 5 mV/s (Figure S1). Figure 3 shows the FTIR spectrum of (a) PoAP–MEL and (b) oAp. All the FTIR peaks assignment for PoAP–MEL and oAP were listed in Table 1. The typical peaks at 3377 and 3307 ${\mathrm{cm}}^{-1}$ in Figure 3, represented the absorption bands of the asymmetrical and symmetrical N–H stretching vibrations.

The peaks at 1512, 1612 and 1478 ${\mathrm{cm}}^{-1}$ are the characteristic bands of the C=C stretching vibration mode for benzenoid rings. The peak at 1585 ${\mathrm{cm}}^{-1}$ representa the N-H bending in primary amines. The peaks at 1407 and 1216 ${\mathrm{cm}}^{-1}$ could be attributed to the C–O–H deformation vibration and the C–O stretching vibration, respectively. The peaks at 1270 and 1216 ${\mathrm{cm}}^{-1}$ represent the O–H deformation vibration and C–O stretching vibration of phenols, respectively. It was noticeable that the characteristic strong absorption N-H bending at 1593 ${\mathrm{cm}}^{-1}$ could still be observed in the PoAP–MEL spectra. Moreover the absorption bands of the asymmetrical and symmetrical N–H stretching vibrations at 3377 and 3307 ${\mathrm{cm}}^{-1}$ in primary amines turned into N–H stretching vibrations at 3351 cm^−1^ in secondary amines after the electropolymerization. Meanwhile, the O–H deformation vibration and C–O stretching vibration of phenols at 1274 ${\mathrm{cm}}^{-1}\;$and C–O stretching vibration of phenols at 1207 ${\mathrm{cm}}^{-1}$ could also be identified. These peaks represent the functional groups on the surface of PoAP, which was able to bond up with “imprinted” molecules (melamine) to constitute binding sites. The IR spectrum obtained was in accordance with the liner PoAP polymer theory during electropolymerization via N-C bonding process [57,58], which might suggested that the MEL adsorpt and desorpt from the binding sites through hydrogen bond with –OH and –NH. Since the template content was too low to be detected by Fourier transform infrared microspectroscopy, no remarkable peak was observed standing for MEL.

### 3.3. Surface Morphology Characterization by AFM

**Figure 4:**
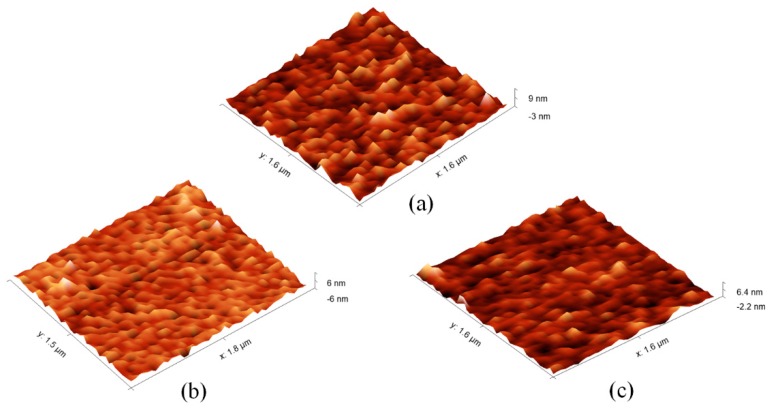
Contact mode 3D AFM images of: (**a**) PoAP–MEL film, (**b**) PoAP–MEL film removed template MEL and (**c**) NIP film. RMS are 1.04 nm, 0.87 nm and 0.61 nm respectively.

AFM was employed to characterize the surface morphology change of PoAP–MEL film before and after template removed. Glass slides (1 cm × 1.5 cm) coated with Au film served as work electrodes. MIP and NIP film samples were synthesized by cyclic voltammetry (30 cycles) in the potential range from –0.3 V to 1.2 V under a scan rate of 50 mV/s. Figure 4 shows typical topographic images by AFM of (a) PoAP–MEL film, (b) PoAP–MEL removed template MEL and (c) NIP film. It reveals that the surfaces were uniform with root-mean-square roughnesses (RMS) of 1.04 nm, 0.87 nm and 0.61 nm respectively. It was quite obvious that PoAP–MEL film was rougher than PoAP–MEL film removed template MEL and NIP film. It was noteworthy that after removed MEL template, the upper surface became flat. Meanwhile the vertical axis revealed that film valleys become much deeper. This might suggest that the binding cavities were exposed. It was speculated that when the imprinted template MEL rebind into the cavities, the film becomes rough, and the effective refractive index increase, leading to a shift in SPR absorption peak. As contrast, the NIP film appeared to be much smoother and thinner than the PoAP–MEL film, and this could be explained by the fact that no MEL template was trapped in the film. Similar features have been reported in previous studies [59,60].

### 3.4. Melamine Detection Based on Resonance Wavelength Modulation

**Figure 5:**
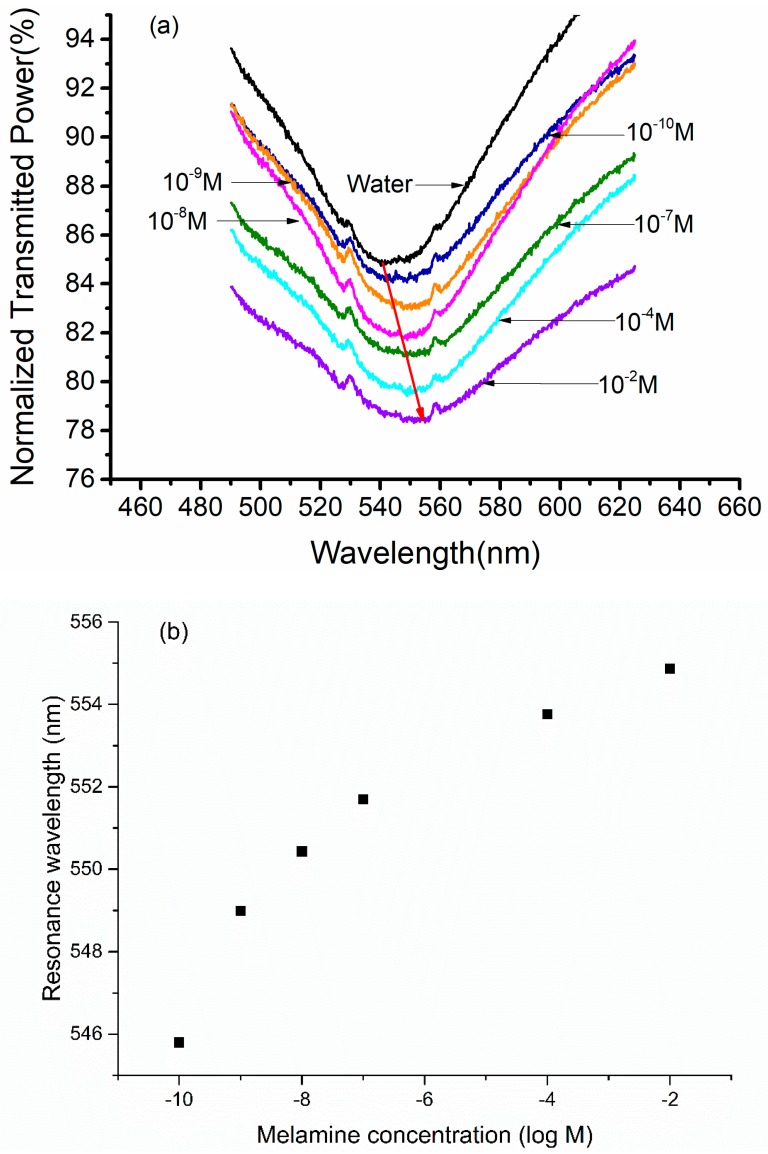
(**a**) SPR spectra for detection of melamine solution with concentration ranging from 0 M to $10^{-2}$ M; (**b**) shift in resonance wavelength for the detection of melamine solution with concentration ranging from 0 M to $10^{-2}$ M.

Melamine solutions of different concentrations ranging from $10^{-10}$ M to $10^{-2}$ M were chosen for the characterization of PoAP–MEL OF-SPR probe. SPR spectrum was recorded immediately after 30 s after each sample solution was injected. Sensing region of the probe was cleaned with MilliQ water between two consecutive samples to remove the residual molecule from previous solution. The SPR spectrum of the MilliQ water was recorded as a reference. The SPR spectra recorded for different concentrations of melamine are shown in Figure 5a. To avoid overlapping between SPR curves, all SPR spectra are not shown in the figure. 

An evident SPR resonance wavelength shifted towards the higher wavelength with the increase in the concentration of melamine solutions, which could be observed from Figure 5b. As previously studied, when a sample molecule comes near the vicinity of the polymer, the non-template molecule would be randomly absorbed around the surface of MIP film, however the template molecule would bind with one of the binding sites with weak interactions due to their complementary shape and size. The binding of template molecule with polymer causes the change in dielectric nature of the gold layer–MIP interface [61]. This change is recognized by change in resonance wavelength in the SPR spectrum since the position of resonance wavelength depends upon the dielectric constant of the polymer. Hence the peak shift in resonance wavelength of SPR represented the melamine molecule amount absorbed onto the binding sites of the PoAP–MEL film [43]. Since the adsorption and desorption kept dynamic equilibrium under thermal equilibrium condition, the shift distance of each SPR resonance wavelength reflected the melamine concentration in each solution.

For melamine concentration of $10^{-10}$ M, resonance wavelength obtained was 545.80 nm while that for $10^{-2}$ M was 554.87 nm. This confirmed a red shift of 9.07 nm in the resonance wavelength for the change in concentration from 10^−10^ M to 10^−2^ M. As a reference, the resonance wavelength obtained for MilliQ water was 539.91 nm, which meant a total 14.96 nm resonance wavelength shift was obtained in 30 s incubation time for each sample. 

However, it can be noted from Figure 5b that the resonance wavelength shift tended to slow down at higher concentrations of melamine. This could be explained by the limited number of binding sites on the MIP film. At lower concentration, the number of binding sites far surpassed the total melamine molecules in solution sample. Most melamine molecules could be absorbed onto the surface of MIP film, which would cause a maximum shift. As the melamine concentration increased, the binding sites started getting crowed, and quite a number of binding sites were filled. The remaining binding sites and the melamine molecules in solution sample maintained dynamic equilibrium, which resulted in a lower shift in resonance wavelength, and appeared a saturation trend.

**Figure 6:**
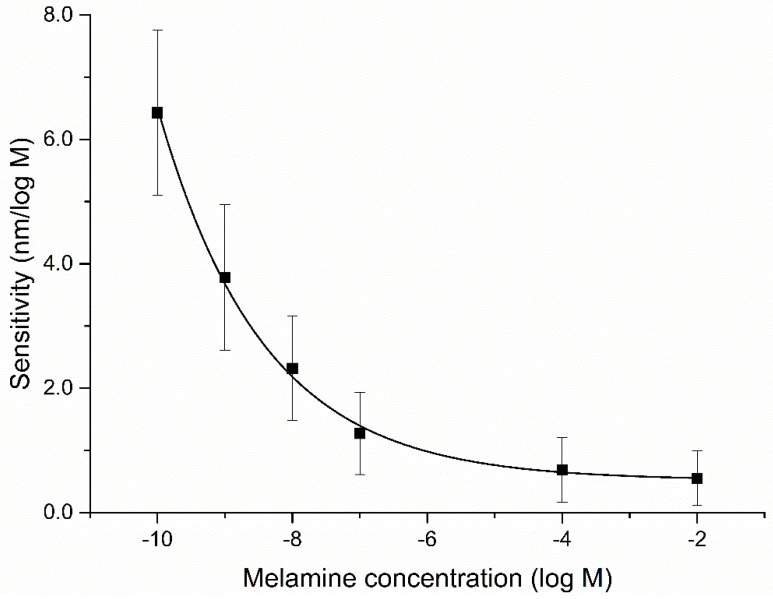
Sensitivity of PoAP–MEL OF-SPR probe for the detection of melamine solution with concentration ranging from 0 M to $10^{-2}$ M.

According to the definition of sensitivity under resonance wavelength modulation mode, which is the derivative of the resonance wavelength curve versus refractive index, we calculated the shift in resonance wavelength per unit change in the logarithmic scale of melamine concentration (nm/log M). From Figure 6, maximum sensitivity of the probe has been found to be 5.89 nm/log M at $10^{-10}$ M melamine sample (the same probe in Figure 5). It decreased with the increase in melamine concentration due to the limited number of binding sites available in the sensing layer mentioned above. It was considered that the probes are more valuable for low concentrations of melamine. The error bar showed the maximum probable error in the measurement for 10 different probes.

**Figure 7:**
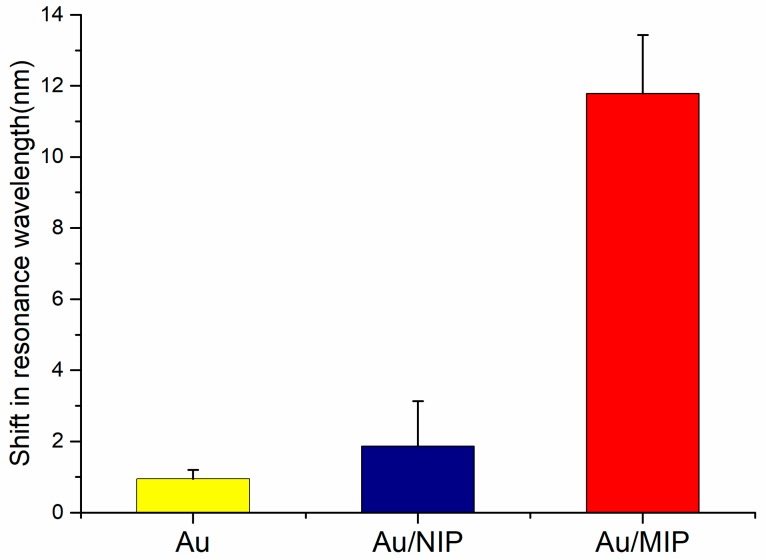
Recognition ability of PoAP–MEL film. Resonance wavelength shift for the detection of melamine solution with concentration ranging from 0 M to $10^{-7}$ M.

To confirm the recognition ability of PoAP–MEL film, resonance wavelength shift of Au/MIP probe was compared with probe covered with bare Au film probe and Au/NIP probe during the detection of melamine solutions with concentration from 0 M to $10^{-7}$ M. All three probes were carefully made to ensure the sensing region and the Au film thickness equal. The comparison of observed shift in resonance wavelength was shown in the histogram (Figure 7). A small shift of 0.95 nm in resonance wavelength was observed for bare Au coated probe since melamine solution concentration from 0 M to $10^{-7}$ M varies little in refractive index. A shift of 1.87 nm in resonance wavelength was observed for Au/NIP coated probe due to barely any binding sites on NIP films. In the case of Au/MIP probe, a remarkable shift of 11.79 nm was observed for the same concentration change of melamine solution. This suggested it is the cavities formed by templates trapped during the polymerization rather than poly(*o*–aminophenol) that had the ability to absorb template molecule.

**Figure 8:**
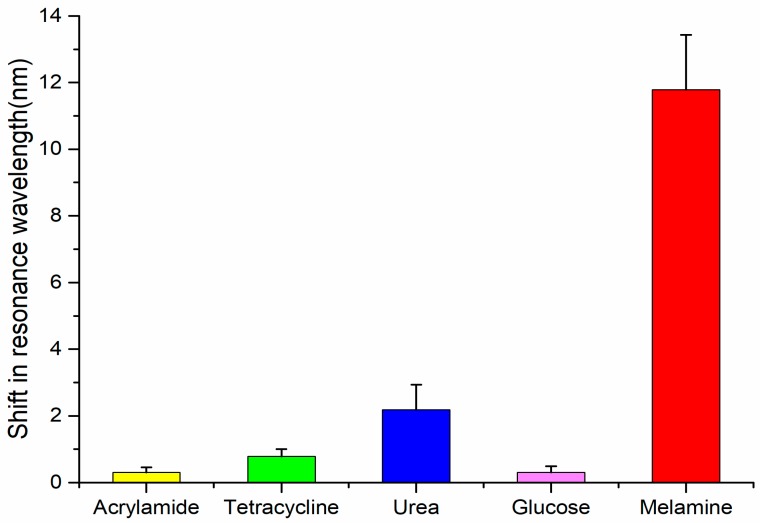
Specificity test of PoAP–MEL film. Resonance wavelength shift for the detection of acrylamide, tetracycline, urea, glucose and melamine solution with concentration ranging from 0 M to $10^{-7}$ M.

Specificity is the fundamental feature of a MIP sensor. In this study, acrylamide, tetracycline, urea, glucose and melamine were chosen to verify the specificity of PoAP–MEL film. Figure 8 shows the comparison of resonance wavelength shift for the change in concentration of analytes from 0 M to $10^{-7}$ M. Compared to other analytes, the observed shift in resonance wavelength reached over 11 nm, which showed a very high specificity for melamine. Meanwhile for acrylamide, tetracycline, urea, glucose they were 0.31 nm, 0.78 nm, 2.18 nm and 0.31 nm respectively. Removal of templates from MIP film exposed cavities with complementary size and shape related to the templates for the subsequent rebinding process, which formed a “lock and key” relationship [62]. Thus, only template molecules could enter these binding sites, while other molecules were unable to form a mass accumulation on the surface of MIP film, which resulted in limited change in dielectric index at the gold layer–MIP interface. Therefore, only little shift in resonance wavelength for other analytes was observed. 

### 3.5. Limit of Detection (LOD)

Limit of detection is another crucial parameter of a sensing device, which represents the detectable analyte concentration near zero concentration. According to Harshit et al. [43], the mathematical expression for LOD is:

(2) $$C_{LOD}=R/S,$$

where $C_{LOD}$ represents the limit of detection of the probe, S stands for the sensitivity near blank (zero) concentration, and R represents spectrometer resolution. Since the spectrometer has a resolution of 0.3 nm and the sensitivity of the probe near zero concentration is 5.89 nm/log M as mentioned above, the calculated value of LOD of the probe is $5.1\times 10^{-12}$ M. To further investigate the performance of the proposed probe, we compared our results with previous studies in Table 2. It could be seen that the probe exhibited remarkable advantages, such as higher sensitivity, wider linear range and lower detection limit. It is worth to note that the LOD in present study is much lower than the safe standard of 2.5 ppm in food products.

## 4. Conclusions

In summary, we have fabricated an OF–SPR probe with PoAP–MEL MIP film synthesized by electropolymerization as sensing layer. An Au film served as the refractive index sensitive metal film coupling with the light leaked out from optical fiber core and the electrode for electropolymerizing MIP film at the same time. *o*-Aminophenol was chosen as functional monomer to trap melamine during the electropolymerizing process. FTIR spectrum revealed that –NH and –OH might be the functional groups bonded up with “imprinted” molecules (melamine) to constitute reactive sites. Surface morphology was characterized by AFM through contact mode. AFM 3D images showed that after template molecules were removed, the upper surface of PoAP–MEL film fattened, while the film valleys became much deeper, suggesting that the binding cavities were exposed. The sensitivity and specificity of the probe were all found to be satisfactory. Compared with previously reported studies, our method had the ability to detect MEL with concentration lower than $10^{-10}\;$M, and had a calculated detection limit of $5.1\times 10^{-12}$ M, which showed significant combined advantages of sensitivity and convenience. Given the low cost, portable size, label–free test, simple preparation process and fast response, this method has potential application to monitor substances in complicated real samples for field tests in the near future.

## Figures and Tables

**Table 1:** FTIR peaks assignment for PoAP–MEL and oAP.

Vibration Assignments for PoAP	Wave Number, $cm^{-1}$
N–H stretching vibrations in secondary amines [51,52,53]	3351 ${\mathrm{cm}}^{-1}$
N-H bending	1593 ${\mathrm{cm}}^{-1}$
C=C stretching of aromatic ring	1501 ${\mathrm{cm}}^{-1}$
O–H deformation vibration and C–O stretching vibration of phenols [52,53]	1274 ${\mathrm{cm}}^{-1}$
C–O stretching vibration of phenols [51]	1207 ${\mathrm{cm}}^{-1}$
Vibration assignments for oAP	Wave number, ${\mathrm{cm}}^{-1}$
N-H bending in primary amines [54,55]	1585 ${\mathrm{cm}}^{-1}$
C=C stretching of aromatic ring	1612 ${\mathrm{cm}}^{-1}$
C=C stretching of aromatic ring [51,53,54,55]	1512 ${\mathrm{cm}}^{-1}$
C=C stretching of aromatic ring [51,55]	1478 ${\mathrm{cm}}^{-1}$
Asymmetrical N–H stretching vibrations [51]	3377 ${\mathrm{cm}}^{-1}$
Symmetrical N–H stretching vibrations [51]	3307 ${\mathrm{cm}}^{-1}$
C–O–H deformation vibrations of phenols [51,53,54,56]	1407 ${\mathrm{cm}}^{-1}$
O–H deformation vibration and C–O stretching vibration of phenols [52,53]	1270 ${\mathrm{cm}}^{-1}$
C–O stretching vibration of phenols [51,52,53]	1216 ${\mathrm{cm}}^{-1}$

**Table 2:** Limit of detection and operating range of various approaches for melamine reported in previous study.

Technique	Operating Range	Limit of Detection
This work	$10^{-10}\;M-10^{-2}\;M$	$5.1\times 10^{-12}\;M$
Impedimetric probe [63]	$1.0\times 10^{-8}\;M-5.0\times 10^{-5\;}M$	$3\times 10^{-9}M$
LC–UV and GC–MSD [11]	–	10 ppb
DAPCI–MS [64]	$10^{-3}-10000{\;\mathrm{mg}\;\mathrm{Kg}}^{-1}$	$3.4\times 10^{-15}{\mathrm{gmm}}^{-2}$
HPLC [12]	–	5 $\;{\mu \mathrm{gg}}^{-1}$
Electrochemical probe [65]	$3.9\times 10^{-8}\;M-3.3\times 10^{-6\;}M$	$9.6\times 10^{-9}\;M$
LC/MS [66]	–	0.008$\;{\mathrm{mg}\;\mathrm{Kg}}^{-1}$
Ion–pair LC–ESI–MS/MS [67]	0.5–100 $\;{\mathrm{ng}\;\mathrm{mL}}^{-1}$	0.01$\;{\mathrm{mg}\;\mathrm{Kg}}^{-1}$
GC–MS and UPLC–MS/MS [68]	1–1000 $\;{\mu g\;\mathrm{mL}}^{-1}$ and 5–1000 $\;{\mu g\;\mathrm{mL}}^{-1}$	10 and 5$\;{\mu g\;\mathrm{Kg}}^{-1}$
GC/MS [14]	0.05–2$\;{\mathrm{mg}\;\mathrm{Kg}}^{-1}$	0.01$\;{\mathrm{mg}\;\mathrm{Kg}}^{-1}$
Electrochemistry (MIP/GCE) [69]	4.0 μM−0.45 mM	$0.36\;\mu M\left(45.4{\;\mathrm{ng}\;\mathrm{mL}}^{-1}\right)$
Electrochemistry (oligonucleotides/Au) [65]	0.039−3.3 μM	$9.6\times 10^{-9}\;M$
MIP/CL [26]	$0.1-50{\;\mu g\;\mathrm{mL}}^{-1}$	0.02$\;{\mu g\;\mathrm{mL}}^{-1}$
Electrochemistry (MIP/potentiometric probe) [61]	5.0 μM−10 mM	1.6 μM
LC [1]	1–400 $\;{\mu g\;\mathrm{mL}}^{-1}$	65$\;{\mu \mathrm{gg}}^{-1}$
HPLC–MS/MS [70]	20–500 $\;{\mathrm{ng}\;\mathrm{mL}}^{-1}$	$5.6{\;\mathrm{ng}\;\mathrm{mL}}^{-1}$
GC–MS/MS [71]	0.04–1.6 $\;\;{\mathrm{mg}\;\mathrm{Kg}}^{-1}$	0.002$\;{\mathrm{mg}\;\mathrm{Kg}}^{-1}$
UV [72]	1.26–10 $\;{\mu g\;\mathrm{mL}}^{-1}$	$0.2{\;\mu g\;\mathrm{mL}}^{-1}$
CZE [73]	–	$0.5{\;\mathrm{mg}\;\mathrm{Kg}}^{-1}$
Electrochemistry (acoustic probe) [74]	5 nM–1 mM	5 nM

## Supplementary Materials

The following are available online at http://www.mdpi.com/1424-8220/18/3/828/s1, Figure S1: CV for the electropolymerization MIP on Au surface. Scan rate: 5 mV/s; number of scans: 150; potential range: −0.4 to 1.3 V.
